# Riverine Microplastic Pollution: Insights from Cagayan de Oro River, Philippines

**DOI:** 10.3390/ijerph20126132

**Published:** 2023-06-15

**Authors:** Aiza D. Gabriel, Ruben F. Amparado, Arnold A. Lubguban, Hernando P. Bacosa

**Affiliations:** 1 Environmental Science Graduate Program, Department of Biological Sciences, Mindanao State University-Iligan Institute of Technology (MSU-IIT), Tibanga, Iligan City 9200, Philippines; aiza.gabriel@g.msuiit.edu.ph (A.D.G.); ruben.amparado@g.msuiit.edu.ph (R.F.A.J.); 2 Premier Research Institute of Science and Mathematics, Mindanao State University-Iligan Institute of Technology (MSU-IIT), Tibanga, Iligan City 9200, Philippines; 3 Department of Chemical Engineering and Technology, Mindanao State University-Iligan Institute of Technology (MSU-IIT), Tibanga, Iligan City 9200, Philippines; arnold.lubguban@g.msuiit.edu.ph; 4 Environmental Pollution and Innovation Laboratory, Center for Sustainable Polymers, Mindanao State University-Iligan Institute of Technology (MSU-IIT), Tibanga, Iligan City 9200, Philippines

**Keywords:** microplastic, river, surface water, Fourier transform infrared spectroscopy, Philippines

## Abstract

Rivers are vital water sources for humans and homes for aquatic organisms. Conversely, they are well known as the route of plastics into the ocean. Despite being the world’s number one emitter of riverine plastics into the ocean, microplastics (MPs), or plastic particles less than 5 mm, in the Philippines’ rivers are relatively unexplored. Water samples were collected from six sampling stations along the river channel of the Cagayan de Oro River, one of the largest rivers in Northern Mindanao, Philippines. The extracted microplastics’ abundance, distribution, and characteristics were analyzed using a stereomicroscope and Fourier transform infrared spectroscopy (FTIR). The results showed a mean concentration of 300 items/m^3^ of MPs dominated by blue-colored (59%), fiber (63%), 0.3–0.5 mm (44%), and polyacetylene (48%) particles. The highest concentration of microplastics was recorded near the mouth of the river, and the lowest was in the middle area. The findings indicated a significant difference in MP concentration at the sampling stations. This study is the first assessment of microplastic in a river in Mindanao. The results of this study will aid in formulating mitigation strategies for reducing riverine plastic emissions.

## 1. Introduction

Plastics advance several societal sectors and improve our quality of life due to their various uses. These include packaging, coatings, adhesives, fabrics, transportation, construction, biomedicine, organic electronics, and nanotechnology [1,2]. Plastic production continues to increase due to its low density, strength, user-friendly design, fabrication capabilities, and, most importantly, low cost [3,4,5]. In addition, the plastic industry has a vital part to play in attaining the Sustainable Development Goals (SDGs) since it generates thousands of job opportunities essential to combatting extreme poverty (SDG 1), which can also impact the other SDGs [6]. However, despite their advantages, plastics are often treated only as disposable commodities, which comes with a significant drawback: waste accumulation [2,6].

When managed appropriately, freshwater ecosystems offer essential ecosystem services to humans such as fish production, water supply, nutrient transport, and recreational value [7,8]. Though the planet comprises about 70% water, only 2.5% is freshwater [9,10]. Freshwater resources on our planet are sparse, but they are, nonetheless, in danger of contamination. According to the United Nations Environment Programme (UNEP), freshwater pollution comes from various sources: power generation, heavy industry, automobiles, municipal, industrial, agricultural, wastewater, fertilizer runoff, and others. Freshwater and marine ecosystems share many similar causes of plastic pollution, including inefficient waste management and improper disposal from residential, industrial, and farming practices, generating plastics that will eventually end up in lakes, rivers, and streams [11]. Additionally, most marine plastics are presumed to be transferred from the land to the ocean via rivers [12,13,14]; however, the plastic pollution in the riverine system in the Philippines is poorly documented.

Plastic pollution is a persistent problem in every country globally, and has increased dramatically for 50 years and impacts living and nonliving ecosystem components [15,16]. In freshwater and marine environments, 80% comes from land-based sources, and the remaining 20% from ocean-based sources such as fishing nets and fishing ropes [17,18]. When exposed to the environment, plastics undergo various weathering processes, which include mechanical fragmentation, photo-degradation, thermal-degradation, and biodegradation [19,20,21,22]. The mechanical and physicochemical properties of these plastics give rise to deterioration and the formation of plastic fragments, known as microplastics (MPs) when their size is less than 5 mm [23,24]. One of the primary sources of plastic pollution in the marine environment is the rivers, which can act as a sink [13,25]. The estimate of the yearly global intake of plastic into the oceans from rivers ranges from 1.15 to 2.41 million tons, with rivers on the Asian continent contributing the majority of this amount [26]. On the other hand, based on the model estimations of Meijer et al. [27], the global annual emissions range between 0.8 million and 2.7 million metric tons per year. With this data, they estimate that there are more than 1000 rivers accounting for 80% of the global annual emission of plastics, wherein the most polluting are those small urban rivers. These model estimations are valuable, yet actual field data are still necessary to validate these findings.

The Philippines is ranked as the third largest emitter of plastic waste into the ocean and the most significant contributor to plastic emissions from riverine sources [27,28]. Plastic litter or macroplastics has been documented in seagrass beds [29], on beaches [30,31,32,33], on seabeds [34], and in riverine systems [35,36]. Persistent plastics can be estimated to degrade over a hundred years; however, they can disintegrate into microplastics (less than 5 mm) and nanoplastics (less than 100 nm) over shorter periods, making it easier for biota to absorb them throughout the food chain [37,38]. Rivers are also prone to the same sources of microplastic as marine habitats, and there is little water for dilution, implying a high concentration of microplastic in the rivers [39]. Most plastic additives in plastics, such as plasticizers, flame retardants, antioxidants, and heavy metals, are not chemically adhered; they can leach and increase the ecotoxicological hazard to aquatic organisms [40,41,42]. Additionally, microplastic contamination has spread to various food products in recent years, directly compromising human health [43,44,45]. Furthermore, they may be potential carriers for spreading bacteria, viruses, organic contaminants, and marine microbes, including antibiotic-resistant bacteria (ARB) [46,47,48]. Despite the serious implications of microplastics, research about them is still in its earliest phases due to methodological and technological constraints [49]. The immediate response was to ban single-use plastic to control the sources of plastic pollution; however, environmental control technology for microplastic is still lacking.

Microplastics can be categorized as primary and secondary microplastics. Primary microplastics, such as those found in facial cleansers and cosmetics, are microscopic when manufactured. Secondary microplastics are produced when huge plastic waste is fragmented through physical, biological, and chemical processes [50,51,52]. Since microplastics are known as emerging contaminants, it has become a concern recently [53]. In the Philippines, microplastics were documented in the marine coastal environment [30,53,54,55,56,57,58], in freshwaters [59,60,61,62], in mangroves [63], in the air [64], and also in the digestive tracts of a variety of organisms [56,65] which conveys the prevalence of microplastics in various environments. Although the Philippines is a prominent plastic polluter from rivers to the ocean [27,35,36,66], the microplastics in rivers are less explored than in the marine environment.

Cagayan de Oro City is a first-class, highly urbanized city in Northern Mindanao, Philippines. It is undergoing rapid commercial, residential, and industrial expansion and is considered an economic hotspot that links agro-based products of neighboring provinces to the country’s central economic institutions in metropolitan cities such as Manila, Cebu, and even abroad [67]. It has a total population of 728,402, with a growth rate of 1.58% (2020 Census of Population and Housing). The Cagayan de Oro River, the study’s focus, is known as the “Whitewater Rafting Capital in the Philippines” since it offers year-round rafting, making it more popular with tourists. It has enormous economic and environmental significance and potential as it serves as a water supply for domestic, industrial, agricultural settings, and for hydroelectric power generation [68]. However, it is also one of five river systems that the country’s Department of Environment and Natural Resources–Environment Management Bureau (DENR–EMB) has identified as biologically threatened [69]. Based on the study undertaken by Meijer et al. [70], the Cagayan de Oro River ranked 32^nd^ (3.6 × 10^3^ metric tons/year) of the top 50 plastic-emitting rivers into the ocean globally. Exposed plastics may degrade into microplastics, and the fact that people who lived close to rivers rely on river life forms, including fish, snails, and plants, for sustenance and household consumption [71] makes it more alarming, highlighting the dire need for this study. However, the extent of microplastic pollution in this ecologically important river has not been systematically studied yet.

In this study, we hypothesized that the lowest microplastic concentrations would be relatively low upstream of the Cagayan de Oro River and higher downstream due to increasing anthropogenic activities. To test this hypothesis, we investigated the abundance, concentration, and distribution of microplastics in the surface water of the Cagayan de Oro River. Specifically, this study aimed to: (a) quantify the microplastic concentrations at the six sampling stations along the Cagayan de Oro River; and (b) determine the color, shape, size, and polymer types of microplastics in the Cagayan de Oro River.

## 2. Materials and Methods

This study was conducted along the Cagayan de Oro River, located in Northern Mindanao. It is part of a river system that drains into the northern central part of Mindanao. The river’s headwaters are situated in the Kalatungan Mountain Range, which is in the central part of the province of Bukidnon [69,72]. It traverses through Talakag, Baungon, and Libona municipalities, filling up tributaries. It finally drains into Macajalar Bay at Cagayan de Oro City in the province of Misamis Oriental [69].

### 2.1. Sampling

**Figure 1:**
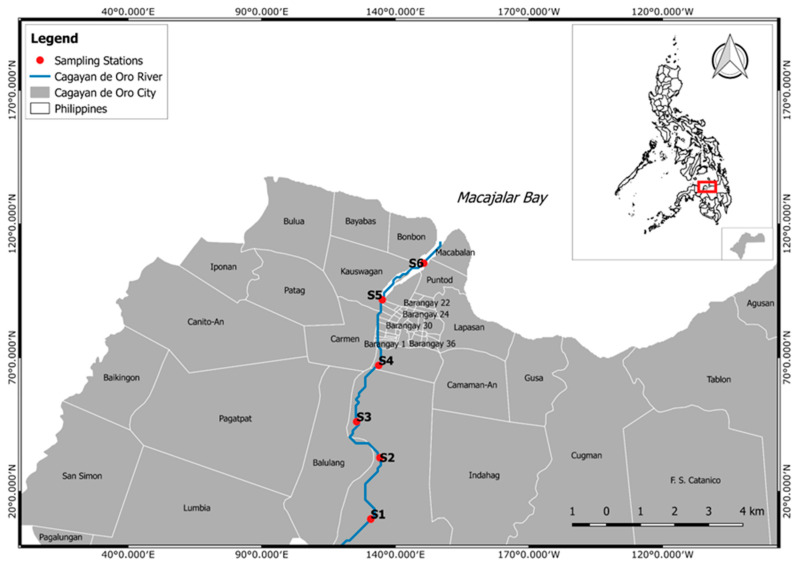
Map of Cagayan de Oro City, Philippines showing the six sampling stations along the Cagayan de Oro River from upstream (S1) to downstream (S6).

This study established six sampling stations, with three sampling points in each station perpendicular to the river channel (Figure 1, Table 1). Two sampling points were established separately within five meters of the riverbanks and one point at the center. Stations 1, 2, and 3 (S1–S3) were established at barangay Macasandig with 3743/km^2^ population density, Station 4 (S4) at barangay Nazareth with 10,002/km^2^ population density, Station 5 (S5) at barangay Kauswagan with 8167/km^2^ population density, and Station 6 (S6) at barangay Puntod with 13,412/km^2^ population density. Barangay Puntod has the highest population density while barangay Macasandig has the lowest. From upstream (S1) to downstream (S6), each station was situated around 1.9 km apart (Figure 1).

We used an inflatable boat to travel to every station. Surface water samples were collected on 15 December 2022, following the methods of Osorio et al. [62] with minor modifications, such as increasing the number of sampling points from one sampling point to three and using a 0.3-mm metal sieve instead of different sizes. Three water samples were collected at each station since there may be variations in microplastic concentration across the channel [73]. A total of 180 L (0.18 m^3^) of water samples were collected across all stations using a stainless steel bucket [62,74], and taking a 10 L (0.01 m^3^) sample from each point at a depth of up to 50 cm. The samples were immediately sieved through a 0.3-mm metal sieve and rinsed with distilled water while being collected in a labeled glass bottle [75,76]. The mouths of the glass bottles were covered with aluminum foil before being sealed with a cap to limit light exposure and contamination. Water samples secured in an ice box were carried to the laboratory and kept in the dark pending further analysis.

### 2.2. Microplastic Extraction and Identification

The sieved samples were placed in a beaker and 10% KOH was added, twice the sample’s volume, to digest the organic matter. The samples were heated to 60 °C for 24 h in the oven. After digestion, the supernatant liquid was vacuum filtered using a 40 mm diameter Whatman GF/C glass filter. The remaining sample with solids underwent density separation for at least 2 h to float the less dense microplastics by adding 30% NaCl solution, twice the volume of the remaining sample. The supernatant carrying microplastics was vacuum filtered again using a Whatman GF/C glass filter. All filter papers used during vacuum filtration were placed in a clean petri dish and dried. The dried filter papers were visually inspected using a stereomicroscope (40× magnification) to classify the suspected microplastics according to color and shape under the criteria set by previous research [77,78]. The suspected microplastics were mounted in between two clean and labeled glass slides using a clean needle and measured afterward. Similar to the method employed by Abidli et al. [79] and Toumi et al. [80], a stereomicroscope with calibrated ocular micrometer was used to measure the size of the suspected microplastics. After microscopy, the suspected microplastics were subjected to Fourier transform infrared spectroscopy (FTIR) analysis (Shimadzu IRTracer-100) to classify polymer type. This method is the most cost-effective way to obtain information regarding particle quantities, polymer types, and sizes [81]. According to Tirkey and Upadhyay [82], FTIR is possibly the best technique for chemical characterization aside from Raman spectroscopy. Compared to Raman spectroscopy with a lower detection threshold (down to 1 μm), FTIR is less time-consuming [81,83]. Additionally, the FTIR method is accurate, does not damage the samples, and produces spectra; however, it has a limited detection threshold that is only feasible for particles up to 20 μm.

### 2.3. Quality Control

Before proper laboratory work, we ensured the laboratory glassware was thoroughly washed and rinsed with distilled water to remove contaminants. Every sample preparation process involved including one control group to confirm the validity of the data collected. Background contamination in the air was also tested through wet filter paper in a petri dish, and no contamination occurred.

### 2.4. Data Analysis

The microplastic concentration was expressed in items per m^3^. The data were first tested with a normality test using the Shapiro–Wilk test. One-way analysis of variance (ANOVA) was used to determine if significant differences were present at the six sampling stations. Tukey’s test for pairwise mean comparisons was used after testing for significance in the ANOVA test at the 95% significance level. Statistical analyses were employed using the PAST software version 4.12b.

## 3. Results

**Figure 2:**
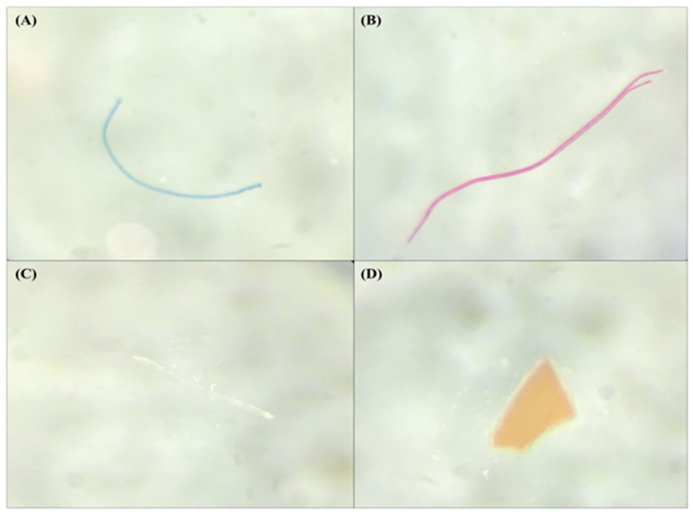
Types of microplastics collected in the surface water of the Cagayan de Oro River, Philippines: (**A**) fiber, S3 (0.7 mm); (**B**) fiber, S2 (0.9 mm); (**C**) filament, S5 (0.5 mm); and (**D**) fragment, S6 (0.4 mm).

All six sampling stations were contaminated with microplastics, which varied in abundance (Table 1). From a total of 90 suspected microplastics that were extracted, 54 (60%) were confirmed to be microplastics after FTIR analysis. Across all the stations, we recorded a mean concentration of 300 items/m^3^ MPs in the surface water of the Cagayan de Oro River. The highest number of MPs with 18 items was at S6, which was close to the mouth of the river, followed by S5 with 17 items, S2 with eight items, S1 with six items, S4 with three items, while the lowest was at S3 (middle) with two items (Table 1). No pattern was observed across all the stations, but it is still evident that the downstream has three times more MPs than were found in the upstream. Figure 2 shows images of the microplastics extracted from the Cagayan de Oro River, along with the stations where they were taken and their sizes. The highest concentration of MPs was detected at S6 (600 ± 57.74 items/m^3^) in barangay Puntod, located downstream near Macajalar Bay. At the station, we observed several pump boats that people use for their livelihood for fishing and transportation, suggesting more anthropogenic activities in the area. Station 6 was situated between two barangays that were connected by a path made possible by the use of pump boats. The second highest concentration was found at S5, located in barangay Kauswagan with 566.7 ± 83.89 items/m^3^, followed by S2 located in barangay Macasandig with 266.7 ± 50.92 items/m^3^, S1 located also in barangay Macasandig with 200 ± 66.67 items/m^3^, and S4 located in barangay Nazareth with 100 ± 57.74 items/m^3^. Meanwhile, the lowest concentration of MPs was found at S3 (middle), located in barangay Macasandig, with 66.7 ± 19.25 items/m^3^. Station 3 was observed to have only a few settlements near the river and was not accessible compared to the other stations. One-way analysis of variance (ANOVA) showed a significant difference (*p* < 0.05) present at the six sampling stations along the Cagayan de Oro River, Philippines. Through the Tukey test (*p* < 0.05), we confirmed that S5 (566.7 ± 83.89 items/m^3^) and S6 (600 ± 57.74 items/m^3^) were significantly higher than S3 (66.7 ± 19.25 items/m^3^) and also S6 (600 ± 57.74 items/m^3^) was significantly higher compared to S4 (100 ± 57.74 items/m^3^). Conversely, there is no significant difference between S6 and S1; however, it is observed that S6 has three times more MP concentration compared to S1, suggesting that there are more MPs downstream than upstream.

**Figure 3:**
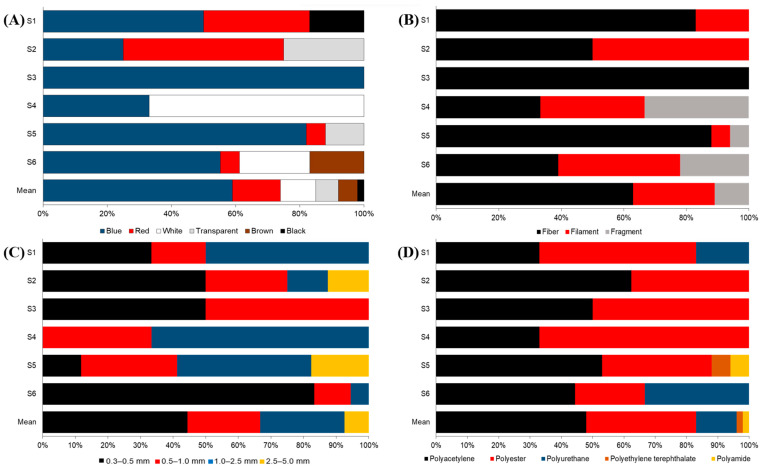
Relative abundance of microplastics in the surface water of the Cagayan de Oro River, Philippines based on (**A**) color, (**B**) shape, (**C**) size, and (**D**) polymer type at different sampling stations.

Extracted MPs were documented and classified based on color, shape, size, and polymer types. As shown in Figure 3, dominant MPs in the Cagayan de Oro River based on the four properties are blue-colored (59%), fiber (63%), 0.3–0.5 mm (44%), and polyacetylene (48%). Blue-colored MP has the highest concentration of 177.8 items/m^3^, accounting for 59% of the total MPs. Red-colored MP has the second highest concentration of 44.4 items/m^3^, accounting for 15% of the total MPs, and is comparable to white-colored MPs with 33.3 items/m^3^, accounting for 11% of the total MPs. Transparent and brown-colored MPs are comparable, with concentrations of 22.2 items/m^3^ (7%) and 16.7 items/m^3^ (6%), respectively. The lowest concentration is black-colored MPs, with only 5.6 items/m^3^ accounting for 2% of the total MPs. We observed that blue and red MPs were present at all stations. Conversely, black-colored MPs were only observed at S1.

**Figure 4:**
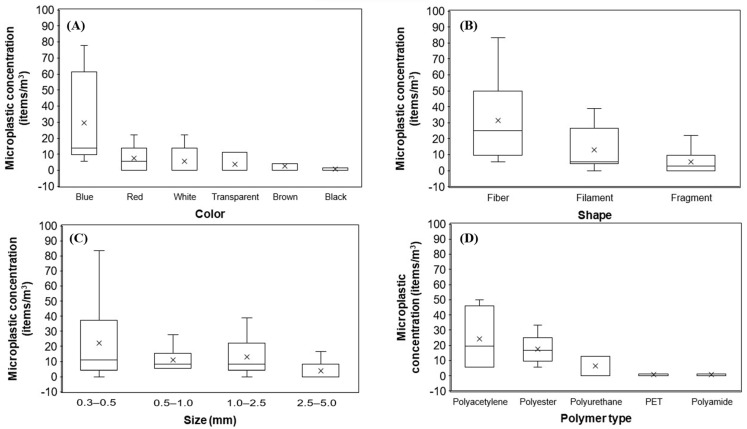
Microplastic distribution at each sampling station based on (**A**) color, (**B**) shape, (**C**) size, and (**D**) polymer type.

**Figure 5:**
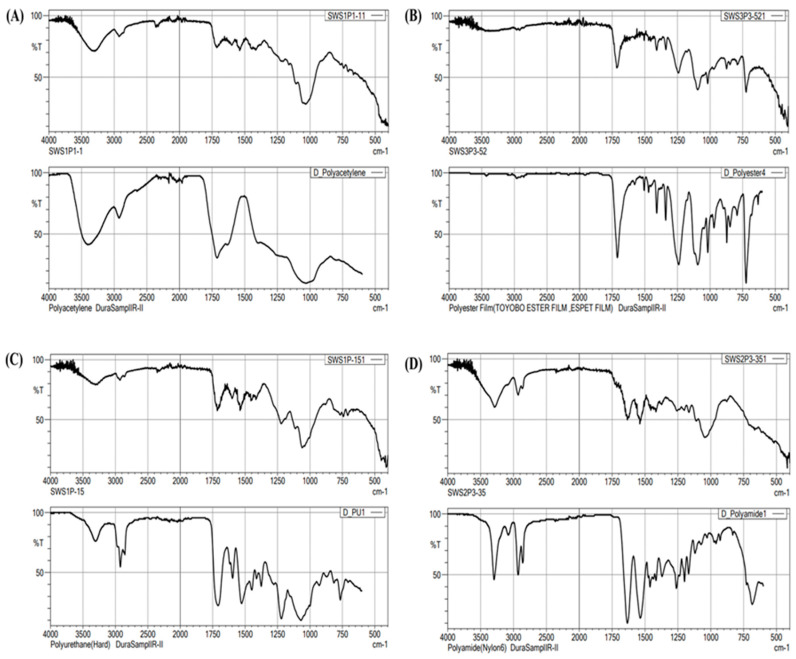
Typical FTIR spectra of collected microplastics, (**A**–**D**) (**top**) and standard microplastics, (**A**–**D**) (**bottom**).

Figure 3B and Figure 4B show that fibers have the highest concentration, totaling 188.9 items/m^3^ and accounting for 63% of the total MPs. The filament has the second highest concentration, with 77.8 items/m^3^ accounting for 26% of the total. In addition, the filament is about two times more than the lowest concentration, fragment (33.3 items/m^3^), accounting for 11% of the total MPs. Microplastics were also classified into four size fractions: 0.3–0.5 mm, 0.5–1.0 mm, 1.0–2.5, and 2.5–5.0 mm (Figure 3C and Figure 4C). The most prevalent sizes were 0.3–0.5 mm with 133.3 items/m^3^ accounting for 44%, followed by 1.0–2.5 mm with 77.8 items/m^3^ (26%), 0.5–1.0 mm with 66.7 items/m^3^ (22%), and 2.5–5.0 mm with 22.2 items/m^3^ (7%). On the other hand, FTIR results confirmed five polymer types in this study: polyacetylene, polyester, polyurethane, polyethylene terephthalate, and polyamide (Figure 5). Their concentrations are 144.4 items/m^3^, 105.6 items/m^3^, 38.9 items/m^3^, 5.6 items/m^3^, and 5.6 items/m^3^, accounting for 48%, 35%, 13%, 2%, and 2%, respectively. Polyacetylene has the highest concentration found present at all sampling stations. Polyester has the second highest concentration and was also present at all stations. On the other hand, polyurethane was only identified at S1 and S6, while polyethylene terephthalate and polyamide were only identified at S5.

## 4. Discussion

We identified that the microplastic concentrations at S6 and S5, located downstream near the mouth of the river, were significantly higher than at S3, which was situated in the middle, and S6 was also significantly higher than S4. There was no significant difference between S6 and S1; however, S6 (downstream) had three times more microplastic than S1 (upstream). The results were similar to the study conducted by Tang et al. [84] in the Songhua River, China, and Buwono et al. [85] in the Brantas River, Indonesia, which also observed that there were higher microplastic concentrations downstream than upstream due to human interference and possible accumulation at the river mouth. Moreover, according to Requiron and Bacosa [35], the closer the sampling point is to the river mouth, the higher the possibility of accumulating plastic litter.

We also observed that the accumulation of microplastic is related to population density since S6, located in barangay Puntod with the highest population density, also has the highest microplastic concentration. In contrast, S3, located in barangay Macasandig with the lowest population density, accumulates the lowest microplastic concentration. Based on the study conducted by Ta and Babel [86], there is a direct correlation between population density and microplastic abundance in the river. In relation to this, anthropogenic activities are closely related to microplastic contamination [87]. At S6, a residential area, the river portion is used for various purposes, including fishing and transportation via pump boat to nearby barangays. Conversely, S3, a non-residential area, has no intensive anthropogenic activities observed.

Compared to other studies, the mean concentration in the Cagayan de Oro River, which is 300 items/m^3^, is considered the lowest among the rivers documented in the Philippines (Table 2). It is also expected since the Pasig River, Parañaque River, Meycauayan River, and Tullahan River were listed as top plastic emitters based on the study conducted by Meijer et al. [27]. Compared to other rivers in China, our study is higher than the study conducted by Mai et al. [88] at the Pearl River Delta and lower than that of Lin et al. [89] along the Pearl River. These variations may be due to methodological approach differences, such as using different mesh or pore sizes during sample collection. Smaller mesh or pore size can collect smaller plastic particles, thereby higher microplastic concentration. In this study, we used a 0.3 mm mesh sieve. In comparison with the other studies with similar mesh or pore sizes, such as the studies conducted along the Gave de Pau River (France), the Surabaya River (Indonesia), the Ofanta River (Italy), and the Pearl Delta River (China), the Cagayan de Oro River has the highest microplastic concentration suggesting regional variation depending on plastic emissions.

Extracted microplastics were classified based on color, shape, size, and polymer type. These properties are potentially relevant to their ecotoxicity; however, changes still depend on environmental conditions and degradation pathways [94].

Blue-colored microplastic was the most dominant color found in the study, which was consistent with the findings of Arcadio et al. [60] in Laguna de Bay, Navarro et al. [63] in Butuan Bay, and Sajorne et al. [30] in Puerto Princesa, Palawan. Previous research revealed that blue-colored MPs were one of the colors highly associated with ropes, safeguard lines, and fishing materials [95,96]. In addition, sources of blue MPs could be linked to plastic and packaging products with long lifespans [47]. Usually, colored MPs are commonly used in packaging, clothing materials, and many other applications [97]. Consequently, studies found that fish seem to mistakenly ingest blue microplastics similar to their copepod prey [98,99]. Ingestion of MPs by aquatic organisms starts harmful effects on gut microbiota [100,101]. A recent study in China showed that various aquatic organisms in the area had been found to consume an average number of MPs ranging from 0.07 particles to 164 particles per individual in different organisms [100]. According to Egbeocha et al. [102], microplastic ingestion can cause endocrine disorders in adult fish, which leads to neoplasia via epigenetic programming. By identifying colors, sources of MPs may also be determined [97].

In terms of shape, the Cagayan de Oro River, Philippines, was dominated by fiber MPs, similar to the study conducted along the Pearl River, China, which accounted for 94.4% of the total [89]. In addition, microplastic studies in the Philippines such as the study undertaken by Arcadio et al. [60] in Laguna de Bay, Navarro et al. [63] in Butuan Bay, and Sajorne et al. [30] on Puerto Princesa Beach, Palawan were all dominated by fiber MPs. One of the causes of the release of fiber MP is washing a garment that can shed more than 1900 fibers per wash, releasing more than 100 fibers per liter of effluent [55]. Fish cages, fishing equipment, and nylon rope deterioration are also significant sources of MP fibers [96]. Studies have shown several negative impacts of MP fibers on aquatic organisms, such as tissue damage, reduced growth, severe intestinal toxicity, and even mortality [103,104]. This can lead to a reduced fish catch, affecting the livelihoods of the local populations, or worse, microplastic-contaminated fish could be eaten by humans.

The most dominant size range of MPs found in the Cagayan de Oro River, Philippines was 0.3–0.5 mm. This is comparable to the study conducted by Faulstich et al. [105] in the sediments of the Namibian River, Namibia where this size range comprised 33.7% of the total MPs. In addition, the study conducted in the Tamsui River also identified 0.3–5 mm size range particles [106]. Different size ranges of MPs represent different pathways for MP exposure and impacts on organisms [107].

Polyacetylene was present at all six sampling stations established in the study area. Recent studies also found polyacetylene in rivers and other environments [108,109,110,111]. It is a conductive polymer that is used as a doping agent for manufacturing various electronic devices due to its thin and stretchable characteristics [112]. Polyester, found in the second-highest level of abundance in the study, was also found at all stations. During the fieldwork, we observed locals washing their clothes in the river. It can be attributed to the presence of polyester since previous research revealed that even delicate hand washing of clothes releases microfiber polyester [113].

Aside from anthropogenic activities, variations in levels of microplastics may be affected by other factors. The previous study confirmed that rainfall significantly increased microplastic concentration [114]. Days before our sampling, rainfall occurred, possibly affecting the MP concentration in the area, and this needs further investigation. Aside from that, hydrodynamic conditions significantly affected the microplastic distribution through the construction of water conservancy hubs and changes in the river’s width [115]. We noticed a higher MP concentration at the stations with wider river widths and vice versa.

## 5. Conclusions

Our study revealed that all six sampling stations along the Cagayan de Oro River, Philippines, were contaminated with microplastics. Higher MP concentrations were observed at Station 6, located downstream near Macajalar Bay, compared to stations found upstream and midstream. Aside from the anthropogenic activities, we also observed that river width affects MPs concentration. The largest river width was identified as having the highest concentration of MPs and vice versa. This study imparts the first documented evidence of the abundance, distribution, and composition of microplastics in the surface water of the Cagayan de Oro River, Philippines, and the first among the rivers in Mindanao, the second largest island in the Philippines. The river is an essential resource for humans and aquatic organisms. Determining pollutants such as microplastics or having baseline data such as documented in the current study will help in achieving the Sustainable Development Goal (SDG) 6, or clean water and sanitation, by informing people, especially policymakers and stakeholders, to take immediate action. This data will help them create new prevention strategies or update existing policies guided by scientific evidence. Furthermore, re-evaluation and stringent enforcement of plastic legislation and the imposition of fines for illegal dumping are encouraged to minimize plastic accumulation, plastic degradation, and detrimental effects on aquatic organisms as well as on humans. Aside from immediate action by policymakers and stakeholders, residents may help address the increasing plastic waste problem by using biodegradable alternatives to plastics and adhering to basic waste management and disposal practices. Adherence to plastic ordinances, such as the recently passed “Single-Use Plastic Ban”, to prevent additional plastic being transported to rivers and other aquatic environments will be highly beneficial.

## Figures and Tables

**Table 1:** Geographical coordinates and characteristics of sampling stations along the Cagayan de Oro River, Philippines, with the number of microplastics and their mean concentration.

Sampling Station	Sampling Point	Coordinates	Characteristics	No. of MPs	Mean Concentration (Items/m^3^ ± SD)
S1	P1 P2 P3	8.429735 N, 124.638408 E 8.429853 N, 124.638133 E 8.430017 N, 124.637802 E	Fishing, Recreational	2 4 0	200 ± 66.67 ^abc^
S2	P1 P2 P3	8.446211 N, 124.640646 E 8.446159 N, 124.640544 E 8.446132 N, 124.640422 E	Fishing, Recreational	1 3 4	266.7 ± 50.92 ^abc^
S3	P1 P2 P3	8.455706 N, 124.634622 E 8.455736 N, 124.634365 E 8.455706 N, 124.634140 E	Non-residential	0 1 1	66.7 ± 19.25 ^c^
S4	P1 P2 P3	8.470570 N, 124.64066 E 8.470710 N, 124.64024 E 8.470918 N, 124.63982 E	Non-residential	0 0 3	100 ± 57.74 ^bc^
S5	P1 P2 P3	8.488010 N, 124.64183 E 8.488176 N, 124.64121 E 8.488471 N, 124.64058 E	Fishing, Residential	8 3 6	566.7 ± 83.89 ^ab^
S6	P1 P2 P3	8.497271 N, 124.653097 E 8.427902 N, 124.652271 E 8.498613 N, 124.651289 E	Fishing, Residential	7 7 4	600 ± 57.74 ^a^

**Note:** Different superscripts in the last column indicate significant differences (*p* < 0.05, Tukey’s test).

**Table 2:** Comparison of microplastic concentration in the Cagayan de Oro River compared to other rivers in the Philippines and those in other countries.

Study Area	Mesh Size/Pore Size (mm)	MP Concentration (Items/m^3^)	Reference
Cagayan de Oro River (Philippines)	0.3	300	This study
Cañas River (Philippines)	0.075	1580	Osorio et al. [62]
Pasig River (Philippines)	0.075	3405	Osorio et al. [62]
Parañaque River (Philippines)	0.075	5015	Osorio et al. [62]
Tullahan River (Philippines)	0.075	11,475	Osorio et al. [62]
Meycauayan River (Philippines)	0.075	57,665	Osorio et al. [62]
Gave de Pau River (France)	0.33	3.26	Bruge et al. [90]
Surabaya River (Indonesia)	0.333	1.47–43.11	Lestari et al. [91]
Ofanto River (Italy)	0.333	0.9–13	Campanale et al. [92]
Pearl River Delta (China)	0.33	0.005–0.7	Mai et al. [88]
Pearl River (China)	0.02	379–7924	Lin et al. [83]
Yellow River (China)	0.05	5358–595,270	Liu et al. [93]

## Data Availability

Not applicable.
